# Inhibition of Intermediate-Conductance Calcium-Activated K Channel (KCa3.1) and Fibroblast Mitogenesis by α-Linolenic Acid and Alterations of Channel Expression in the Lysosomal Storage Disorders, Fabry Disease, and Niemann Pick C

**DOI:** 10.3389/fphys.2017.00039

**Published:** 2017-01-31

**Authors:** Aida Oliván-Viguera, Javier Lozano-Gerona, Laura López de Frutos, Jorge J. Cebolla, Pilar Irún, Edgar Abarca-Lachen, Ana J. García-Malinis, Ángel Luis García-Otín, Yolanda Gilaberte, Pilar Giraldo, Ralf Köhler

**Affiliations:** ^1^Biomedical Signal Interpretation and Computational Simulation Group, Aragón Institute for Engineering Research (I3A), University of ZaragozaZaragoza, Spain; ^2^Instituto de Investigación Sanitaria AragónZaragoza, Spain; ^3^Biomedical Research Networking Center in Bioengineering, Biomaterials and NanomedicineZaragoza, Spain; ^4^Aragón Institute of Health SciencesZaragoza, Spain; ^5^Departamento de Bioquímica, Biología Molecular y Celular, Facultad de Ciencias, Universidad de ZaragozaZaragoza, Spain; ^6^Spanish Foundation for the Study and Treatment of Gaucher Disease and Other Lysosomal DisordersZaragoza, Spain; ^7^Centro de Investigación Biomédica en Red de Enfermedades RarasZaragoza, Spain; ^8^Faculty of Health Sciences, Universidad San JorgeVillanueva de Gállego, Spain; ^9^Service of Dermatology, Hospital San JorgeHuesca, Spain; ^10^Aragón Agency for Research and DevelopmentZaragoza, Spain

**Keywords:** KCa3.1/SK4 channel, linolenic acids, fabry disease, fibroblasts, ion channel pharmacology

## Abstract

The calcium/calmodulin-gated KCa3.1 channel regulates normal and abnormal mitogenesis by controlling K^+^-efflux, cell volume, and membrane hyperpolarization-driven calcium-entry. Recent studies suggest modulation of KCa3.1 by omega-3 fatty acids as negative modulators and impaired KCa3.1 functions in the inherited lysosomal storage disorder (LSD), Fabry disease (FD). In the first part of present study, we characterize KCa3.1 in murine and human fibroblasts and test the impact of omega-3 fatty acids on fibroblast proliferation. In the second, we study whether KCa3.1 is altered in the LSDs, FD, and Niemann-Pick disease type C (NPC). Our patch-clamp and mRNA-expression studies on murine and human fibroblasts show functional expression of KCa3.1. K_Ca_ currents display the typical pharmacological fingerprint of KCa3.1: Ca^2+^-activation, potentiation by the positive-gating modulators, SKA-31 and SKA-121, and inhibition by TRAM-34, Senicapoc (ICA-17043), and the negative-gating modulator, 13b. Considering modulation by omega-3 fatty acids we found that α-linolenic acid (α-LA) and docosahexanenoic acid (DHA) inhibit KCa3.1 currents and strongly reduce fibroblast growth. The α-LA-rich linseed oil and γ-LA-rich borage oil at 0.5% produce channel inhibition while α-LA/γ-LA-low oils has no anti-proliferative effect. Concerning KCa3.1 in LSD, mRNA expression studies, and patch-clamp on primary fibroblasts from FD and NPC patients reveal lower KCa3.1-gene expression and membrane expression than in control fibroblasts. In conclusion, the omega-3 fatty acid, α-LA, and α-LA/γ-LA-rich plant oils, inhibit fibroblast KCa3.1 channels and mitogenesis. Reduced fibroblast KCa3.1 functions are a feature and possible biomarker of cell dysfunction in FD and NPC and supports the concept that biased lipid metabolism is capable of negatively modulating KCa3.1 expression.

## Introduction

The intermediate-conductance calcium/calmodulin-gated potassium channel KCa3.1 (encoded by the *KCNN4* gene) is expressed in a variety of tissues such red and white blood cell lineage, epithelia, and endothelia (Wei et al., 2005). Notably, induction of KCa3.1 is suspected to drive abnormal cell proliferation, inflammation, and pathological organ remodeling (fibrosis) in heart, kidneys, lungs, some cancers, and atherosclerosis and neo-angiogenesis (for review see: Roach et al., 2013; Feske et al., 2015; Huang et al., 2015; Köhler et al., 2016). Accordingly, pharmacological modulation of the channel is considered a way to treat disease in humans. Hence, several small molecule blockers and positive and negative-gating modulators are available and Icagen compound, Senicapoc, was found clinically safe (for recent review see: Christophersen and Wulff, 2015). Also the endogenous omega-6 fatty, arachidonic acid (AA) and AA-metabolites such as some 11,12 epoxyeicosatrienoic acid and 20-hydroxyeicosatetraenoic acid (20-HETE), have been found to inhibit cloned human KCa3.1 that required mechanistically the inner pore lining amino acid residues, T250 and V275 as putative interaction sites (Hamilton et al., 2003; Kacik et al., 2014). However, the cell biological meaning of this modulation of KCa3.1 by omega-3/6 fatty acid is unclear.

Interestingly, KCa3.1-functions have been suggested to be compromised in Fabry disease (FD; Choi et al., 2014, 2015; Choi and Park, 2016), a X-linked lysosomal storage disorder (LSD), in which defective lysosomal targeting of mutated α-galactosidase A encoded by the *GLA* gene causes globotriaosyl-ceramide (Gb3) accumulation. Mechanistically, Gb3 has been suggested to downregulate KCa3.1-expression, fibroblast growth, differentiation into myofibroblasts, and collagen expression. These impairments could be reversed by channel activation (Choi et al., 2015).

In keeping with these previous findings, we characterized in the first part of this study KCa3.1 functions in a murine fibroblast cell line and human dermal fibroblasts and tested inhibitory actions of α-linolenic acid (α-LA) and docosahexaenoic acid (DHA) on channel functions and fibroblast mitogenesis.

In the second part, we tested whether disease-related impairments of fibroblast KCa3.1 are features of the two LSDs, FD and Niemann-Pick disease type C (NPC).

In this study, we found that murine and human dermal fibroblasts express amounts of functional KCa3.1 channels. We show strong sensitivity of fibroblast mitogenesis to α-LA and DHA.

KCa3.1 gene expression and KCa3.1 currents were reduced in two different LSDs.

## Methods

### Cells and patients

#### Fibroblast cultures

Murine 3T3-L1 fibroblasts were cultured in DMEM supplemented with 10% FCS (Biochrom KG, Berlin, Germany). Primary human dermal fibroblast cultures were made from punch biopsies from FD and NPC patients as well as from a healthy donor (according to the guidelines of the local Ethics Committee, permit no. PI16/0227). Fibroblasts from patients' skin biopsies were cultured in DMEM supplemented with 10% FCS (Biochrom KG, Berlin, Germany) and cells were used at passages 1–4. For patch-clamp experiments, cells were seeded on coverslips and used within 24 h.

This study was carried out in accordance with the recommendations of the local Ethics Committee (CEICA) with written informed consent from all subjects. All subjects gave written informed consent in accordance with the Declaration of Helsinki. The protocol was approved by the local Ethics Committee (CEICA).

#### Patients

The FD patients included in this study displayed the classic FD phenotypes: A 32-years old male (p.Y216fs^*^15 hemizygous) showed acroparesthesias, hypohidrosis, and mild proteinuria and renal glycolipid deposits and frequent gastrointestinal disturbances. The 56-years old female (p.G183V heterozygous) showed despite her heterozygous genotype clear and typical clinical symptoms like acroparesthesias, hypohidrosis, mild proteinuria, and renal glycolipid deposits, cardiac arrhythmias, and frequent gastrointestinal disturbances. An atypical 50-years old female FD patient harbored a complex intronic haplotype (CIH) (c.-10C > T, c.369 + 990C > A, c.370-81_370-77delCAGCC, c.640-16A > G, c.1000-22C > T, heterozygous) (Gervas-Arruga et al., 2015) and showed ischemic coronary artery disease with repetitive balloon catheter inventions, stent implantations, and valve insufficiency. A 41-years old male FD patient with a complex intronic haplotype (CIH) (c.-10C > T, c.369 + 990C > A, c.370-81_370-77delCAGCC, c.640-16A > G, c.1000-22C > T, hemizygous; Gervas-Arruga et al., 2015) who had a different phenotype with mild renal deposits in podocytes and normal glomerular function, but showed severe acroparesthesias and two transitory episodes of ischemic stroke.

The Fabry patients started enzymatic replacement therapy in 2011 with agalsidase alfa weekly and are clinically stable at present.

The 31-years old male NPC patient (*NPC1* p.Q775P heterozygous) did not show neurological manifestation yet. The patient developed severe spleen enlargement with extreme invasion of foam cells and was splenectomized.

### Patch-clamp electrophysiology

In brief, Ca^2+^-activated K^+^ currents were measured in the whole-cell configuration using an EPC10-USB amplifier (HEKA, Electronics, Lambrecht-Pfalz, Germany) and a K pipette solution (intracellular) containing 1 μM Ca^2+^ free (in mM): 140 KCl, 1 MgCl_2_, 2 EGTA, 1.71 CaCl_2_ (1 μM [Ca^2+^]_free_), and 5 HEPES (adjusted to pH 7.2 with KOH). The bath solution contained (in mM): 140 NaCl, 5 KCl, 1 MgSO_4_, 1 CaCl_2_, 10 glucose, and 10 HEPES (adjusted to pH 7.4 with NaOH). For maximal KCa3.1-activation we applied SKA-31 or SKA-121 at a concentration of 1 μM that is ~10-times the concentration for half-maximal activation.

For data acquisition and analysis we used the patch-master program (HEKA). Ohmic leak currents of up to 1 nS were subtracted where appropriate. We quantified outward currents at a 0 mV holding potential (to avoid contamination with non-selective cation currents and chloride currents). Capacitance values (a measure of cell surface and thus cell size) were significantly higher in fibroblasts from female and male FD GLA Intronic hemizygous than in Ctrl (Ctrl, 38 ± 5 pF; ♀ FD *GLA* Intronic heterozygous, 80 ± 12 pF (*P* < 0.05 vs. Ctrl, Student's *T*-Tests); ♂ FD *GLA* Intronic hemizygous, 107 ± 10 pF (*P* < 0.05 vs. Ctrl, Student's *T*-Tests); ♀ FD *GLA* p.G183V heterozygous, 24 ± 5 pF; ♂ FD *GLA* p.Y216fs^*^15 hemizygous, 50 ± 8 pF, *n* = 6; ♂ *NPC1* p.Q775P heterozygous, 34 ± 6 pF, *n* = 5).

### Gene expression studies

#### RNA isolation and reverse transcription

Total RNA was isolated with TriReagent (Sigma, Saint Louis, Missouri, USA) following manufacturer's protocol, and further purified using RNA Clean-up and Concentration-Micro-Elute kit (Norgen Biotek, Thorold, Canada). Genomic DNA digested using the Ambion DNA-free kit (Invitrogen, Carlsbad, California, USA). Quantity and purity of extracted RNA were determined by spectrophotometry (NanoDrop 1000, Thermofisher, Waltham, MA) and stored at −80°C for later use. Integrity of RNA samples and successful digestion of genomic DNA were ensured by gel electrophoresis. Reverse transcription was performed with 600 ng of total RNA by using the SuperScript III reverse transcriptase (Invitrogen, Carlsbad, California, USA) and random hexamers following the manufacturer's protocol.

#### Quantitative RT-PCR

cDNA obtained from 20 ng of total RNA was amplified in triplicates using the SYBR Select Master Mix and a StepOnePlus Real-Time PCR system (Applied Biosystems, Foster City, California, USA) using the following cycle protocol: 95°C, 15 s and 60°C, 60 s repeated for 40 cycles. As final step, a melting curve analysis was carried out to verify correct amplification. The following primers were used: KCa3.1-F: 5′-CATCACATTCCTGACCATCG-3′; KCa3.1-R: 5′-ACGTGCTTCTCTGCCTTGTT-3′, GAPDH-F: 5′-GGGATCAATGACCCCTTCAT-3′; GAPDH-R: 5′-GCCATGGAATTTGCCAT-3.

Data were analyzed with LinRegPCR software (Ruijter et al., 2009) and gene expression levels relative to GAPDH expression as reference gene and normalized to control were calculated using the formula:

$$\%\;of\;Control=\frac{Efficiency{\left(KCa3.1\right)}^{Cq\left(Control\right)-Cq\left(Sample\right)}}{Efficiency{\left(GAPDH\right)}^{Cq\left(Control\right)-Cq\left(Sample\right)}}$$

### Proliferation assay

Measurements of cell proliferation were done using the vital colorimetric Janus-Green assay, with some modifications as described previously in more detail (Olivan-Viguera et al., 2013). In brief, 3T3-L1 fibroblasts were seeded at the same density (80,000 cells/well) in 12-well-plates (flat bottom, Costar, Corning Inc. NY, USA) and cultured in DMEM medium supplemented with L-glutamine, with 10% fetal bovine serum and 1% penicillin/streptomycin, in the presence of DHA (10 μM), α-LA (10–500 μM), borage oil, argan oil, onagra oil, linseed oil (0.5%), or vehicle (DMSO 0.1% for DHA or DMSO 0.5% for α-LA, borage oil, argan oil, onagra oil, and linseed oil). Final DMSO concentrations were the same for each compound and its control. At days 0, 1, 2, 3, and 4 (confluence) cells were fixed with formalin (10% in deionized water). Thereafter, cells were stained for 10 min with 0.3% Janus Green B dye (Sigma-Aldrich) at room temperature at constant stirring. Cells were then de-stained with water and dye was eluted with 0.5 M HCl at room temperature at constant stirring for 15 min. Absorbance values at 595 nm were determined using a microplate reader (Sinergy HT, Biotek, USA). Each compound was tested in three independent experiments. In other experiments, high power field images of human fibroblast cultures were taken at day 0 and 4 and the increase of area occupancy was determined by using the Image J software. We did not find gross differences between controls (increase in area occupancy: 58%) and FD patients (male *GLA* p.Y216fs^*^15 hemi, 57%; female *GLA* p.G183V heterozygous, 86%) and the male *NPC1* p.Q775P heterozygous, 93%).

### Compounds

α-LA and docosahexaenoic acid (DHA) were purchased from Sigma-Aldrich (Deisenhofen, Germany) and were pre-diluted in DMSO giving a concentration of 1–500 μM (final concentration of DMSO 0.1–0.5%). TRAM-34, Senicapoc, SKA-31, and SKA-121 were a kind gift from Prof. Heike Wulff, Pharmacology at University of California, Davis. 13b ([3,5-bis[(3-fluoro-4-hydroxy-benzoyl)-oxymethyl]phenyl]methyl 3-fluoro-4-hydroxy-benzoate) was a kind from Prof. Robert Kiss, Laboratoire de Toxicologie, Institut de Pharmacie, ULB, Belgium. All other drugs were purchased from Sigma-Aldrich (Deisenhofen, Germany). Pure plant oils were purchased from a local herbalist's shop and pre-diluted in DMSO (final concentration of both, 0.5%).

## Results

### Characterization of KCa3.1 in murine 3T3-L1-fibroblasts and impact of the omega-3 fatty acids, α-LA and DHA, on 3T3-L1 mitogenesis *in vitro*

**Figure 1:**
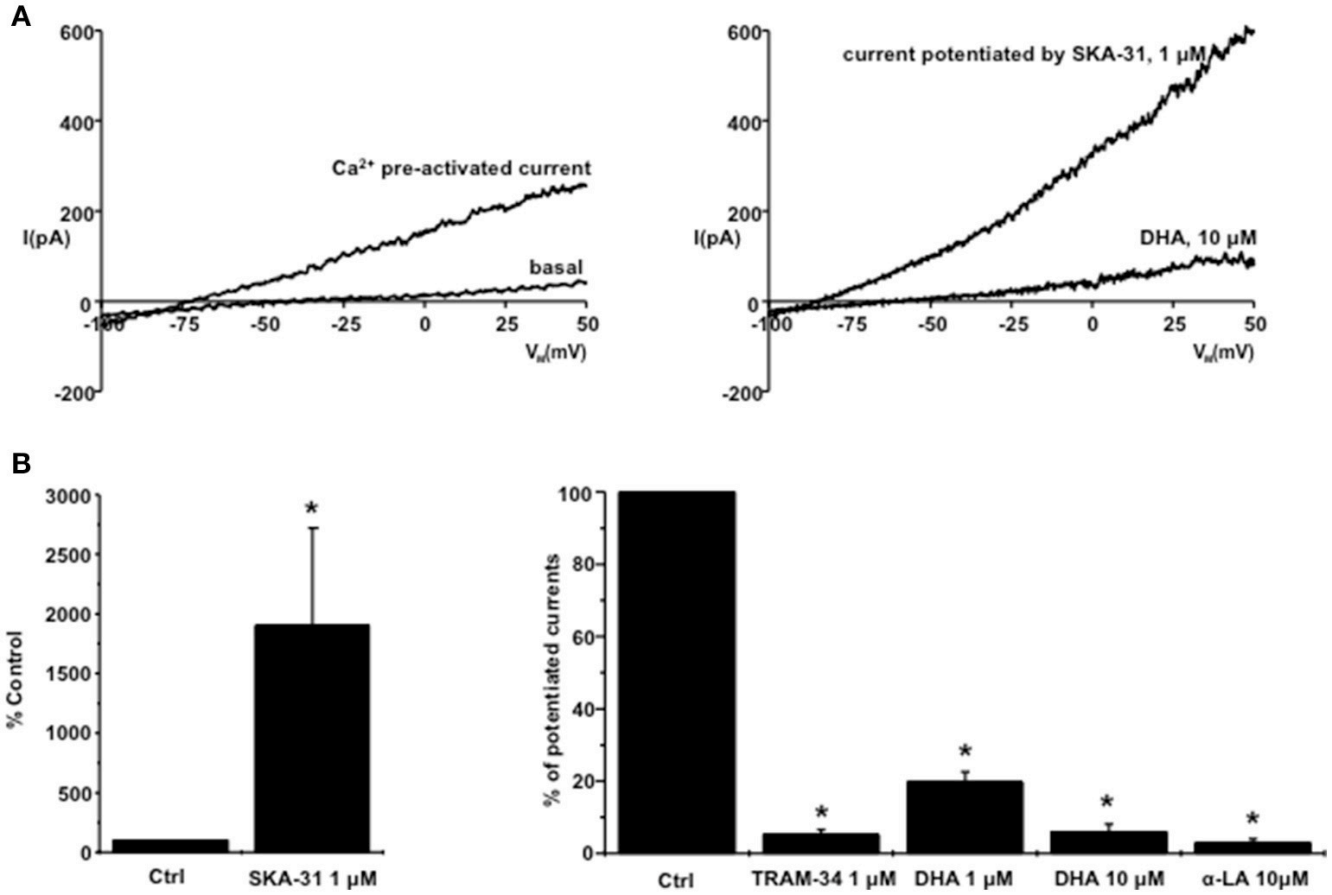
**Inhibition of KCa3.1 in murine 3T3-L1 fibroblasts by DHA and α-LA: (A)** on left: Exemplary whole-cell current recordings of basal currents (after breaking into the whole-cell mode) and of pre-activated KCa3.1-currents by calcium. On right: SKA-31-potentiation of KCa3.1 currents (pre-activated by calcium) and inhibition of the complete potentiated current by DHA. **(B)** Summary data showing potentiation of calcium-pre-activated KCa3.1 currents by SKA-31 and inhibition of the complete SKA-31-potentiated KCa3.1 currents by DHA and α-LA. Data are means ± SEM. ^*^P < 0.05, Student's *T*-test.

In murine 3T3-L1 fibroblasts, KCa3.1 currents developed after breaking into the whole-cell mode and concomitant infusion of Ca^2+^ via the patch-pipette. KCa3.1 currents displayed voltage-independence (for traces see Figure 1A, left panel; for additional details see Oliván-Viguera et al., 2015b). Pre-activation of the KCa3.1 currents and concomitant K+-efflux was accompanied by a shift of the reversal potential (E_rev_) from basal (E_rev_, ≈ −25 ± 7 mV, immediately measured after breaking into the whole-cell mode) to a more negative E_rev_ of ≈ −55 ± 9 mV.

The positive-gating modulators of KCa3.1, SKA-31 (1 μM, Sankaranarayanan et al., 2009) strongly potentiated the calcium-pre-activated currents by 19-fold and further shifted the E_rev_ to ≈ −76 ± 3 mV (for trace see Figure 1A, right panel, and summary data in Figure 1B, left panel). The classical KCa3.1 blocker, TRAM-34 (Wulff et al., 2000), virtually abolished the complete potentiated KCa3.1 currents (Figure 1B, right panel).

Regarding omega-3 fatty acids, α-LA and DHA, both at 10 μM, also abolished the complete SKA-31-potentiated KCa3.1 current and shifted E_rev_ back to more positive values, E_rev_ ≈ −11 ± 4 mV (for trace see Figure 1A and summary data Figure 1B, right panel).

QRT-PCR amplified mRNA-KCa3.1 in murine 3T3-L1 fibroblasts and expression levels were 0.02% of the reference gene, GAPDH, thus demonstrating intact gene expression of KCa3.1 in these cells.

**Figure 2:**
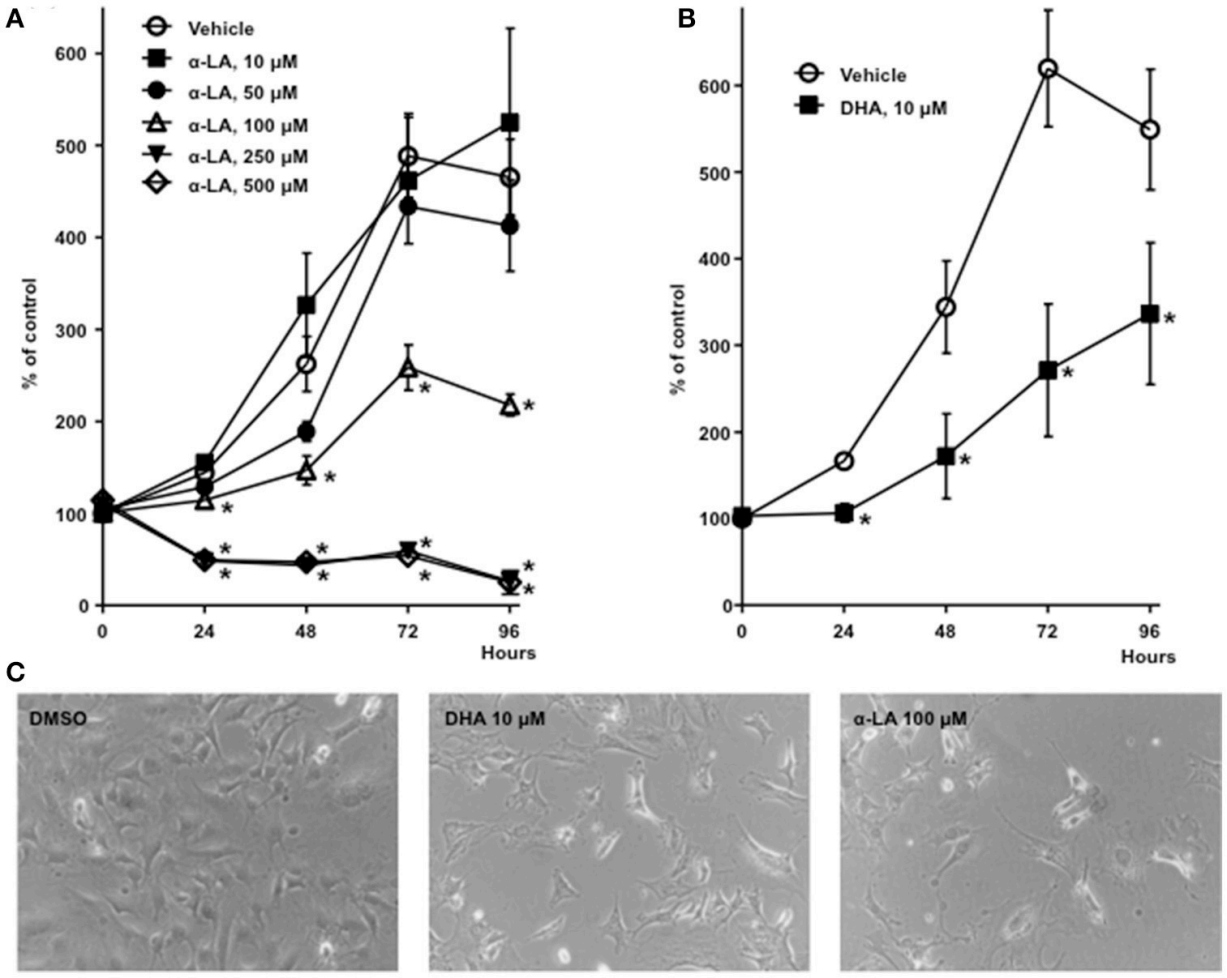
**Inhibition of 3T3-L1 fibroblast mitogenesis by α-LA and DHA: (A)** Concentration-dependent inhibition of cell proliferation by α-LA. **(B)** Inhibition of proliferation by DHA. Data are means ± SEM. ^*^P < 0.05, Student's *T*-tests. **(C)** High power field images of 3T3-L1 fibroblasts after 4 days of culture in the presence of DMSO (vehicle, 0.5%), DHA 10 μM and α-LA 100 μM.

Concerning the impact of α-LA on fibroblast proliferation, our Janus Green B colorimetric assay of cell proliferation revealed that α-LA concentration-dependently inhibited cell growth, with half-maximal inhibition at ≈100 μM (Figures 2A,C). However, concentrations ≥250 μM appeared to be cytotoxic in this assay. We also observed inhibition of cell growth when using DHA (Figures 2B,C), although DHA was seemingly more potent, with half-maximal inhibition at ≈10 μM.

**Figure 3:**
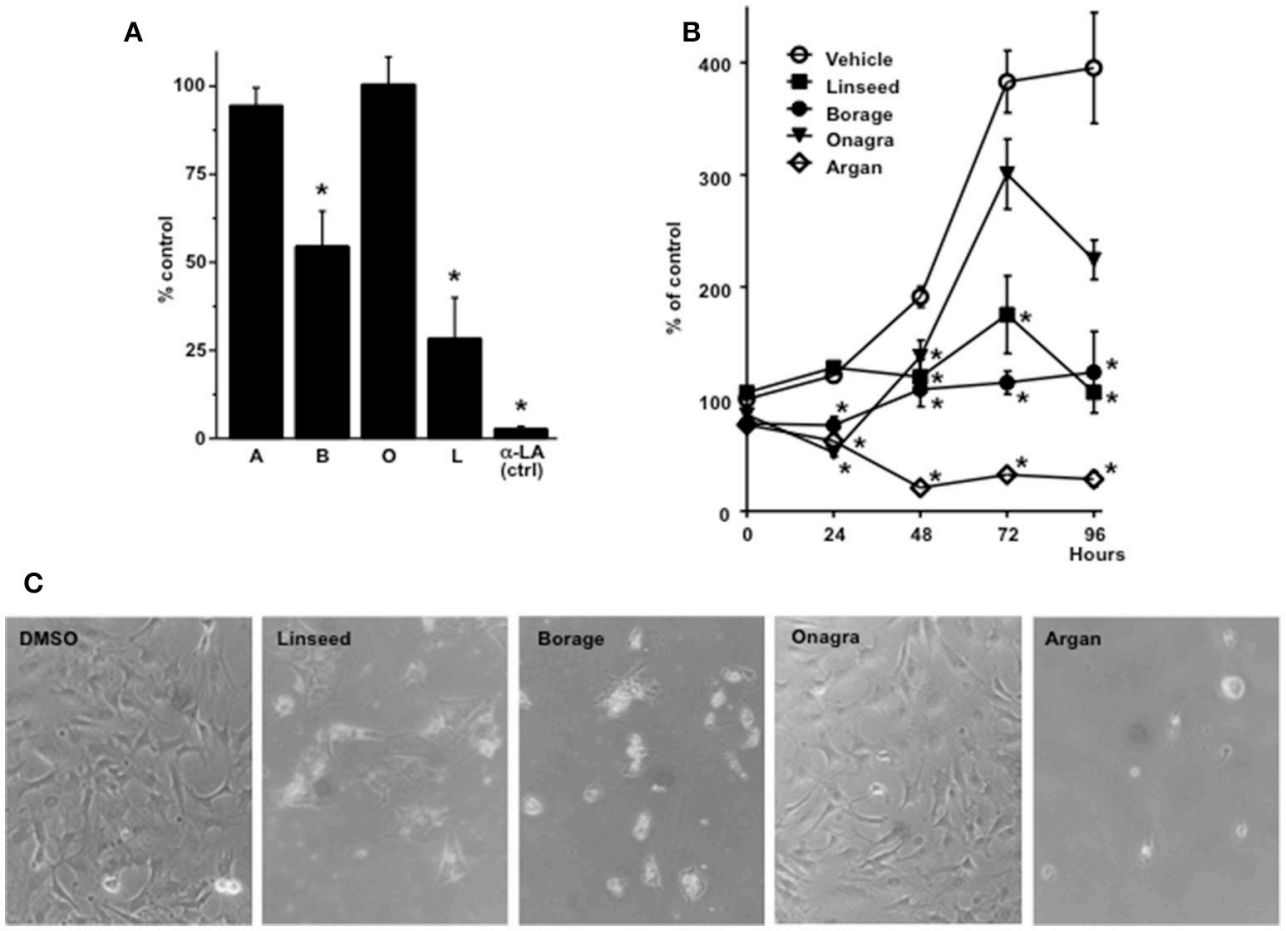
**Inhibition of KCa3.1 currents and 3T3-L1 fibroblast proliferation by plant oils. (A)** Summary data showing the efficacy of different plant oils to inhibit KCa3.1 currents. A, argan oil; B, borage oil; O, onagra oil; L, linseed oil. α-LA at 10 μM served as positive control. The concentration of the oils was 0.5%. **(B)** Efficacy of plant oils to inhibit cell proliferation. Note that argan oil produced a decrease in cell number after 24 and 48 h, which was indicative of high cytotoxicity in this assay. Data are means ± SEM. ^*^P < 0.05, Student's *T*-test. **(C)** High power field images of 3T3-L1 fibroblasts after 4 days of culture in the presence of DMSO (vehicle), linseed oil, borage oil, onagra oil, and argan oil (0.5%).

Considering the reported beneficial effects of dietary supplementation of α-LA and α-LA-rich plant oils on autoimmune diseases and chronic inflammation such as multiple sclerosis, rheumatoid arthritis, psoriasis, chronic inflammatory bowel disease, or atopic dermatitis (Macfarlane et al., 2011; Wergeland et al., 2012; Yates et al., 2014) we were curious to learn whether α-LA-rich plant oils were also capable of inhibiting KCa3.1. The α-LA-rich linseed oil (from *Linum usitatissimum* and γ-LA-rich borage seed oil [*Borago officinalis* (Tso et al., 2012) produced significant channel inhibition at 1% while argan oil (from kernels of the argan tree (*Argania spinosa* L. (Charrouf and Guillaume, 2008) and onagra oil (from evening primrose seeds, *Oenothera biennis* L. (Fan and Chapkin, 1998)] with low levels of α-LA (<1%) and γ-LA (<10%), respectively, did not produce significant channel inhibition (Figure 3A).

Concerning 3T3-L1 fibroblast proliferation, linseed oil and borage oil produced significant inhibition of mitogenesis (Figures 3B,C) while onagra oil showed no significant effect on proliferation. Argan oil was found to be cytotoxic as cell numbers instantaneously decreased after application to cells (Figures 3B,C).

### Characterization of KCa3.1 in cultured human dermal fibroblasts and comparisons of fibroblast KCa3.1 functions in FD and NPC patients

While KCa3.1 in murine fibroblast lines is well-characterized (Olivan-Viguera et al., 2013), information on KCa3.1 function and membrane expression in human dermal fibroblasts is scarce if compared to KCa3.1 in human fibroblasts in other tissues (Roach et al., 2014, 2015; Friebel et al., 2015). Moreover, altered KCa3.1 functions have been described in murine models of FD and FD patients (Choi et al., 2014, 2015; Choi and Park, 2016), suggesting a detrimental impact of lysosomal lipid accumulation on KCa3.1 protein expression or function.

**Figure 4:**
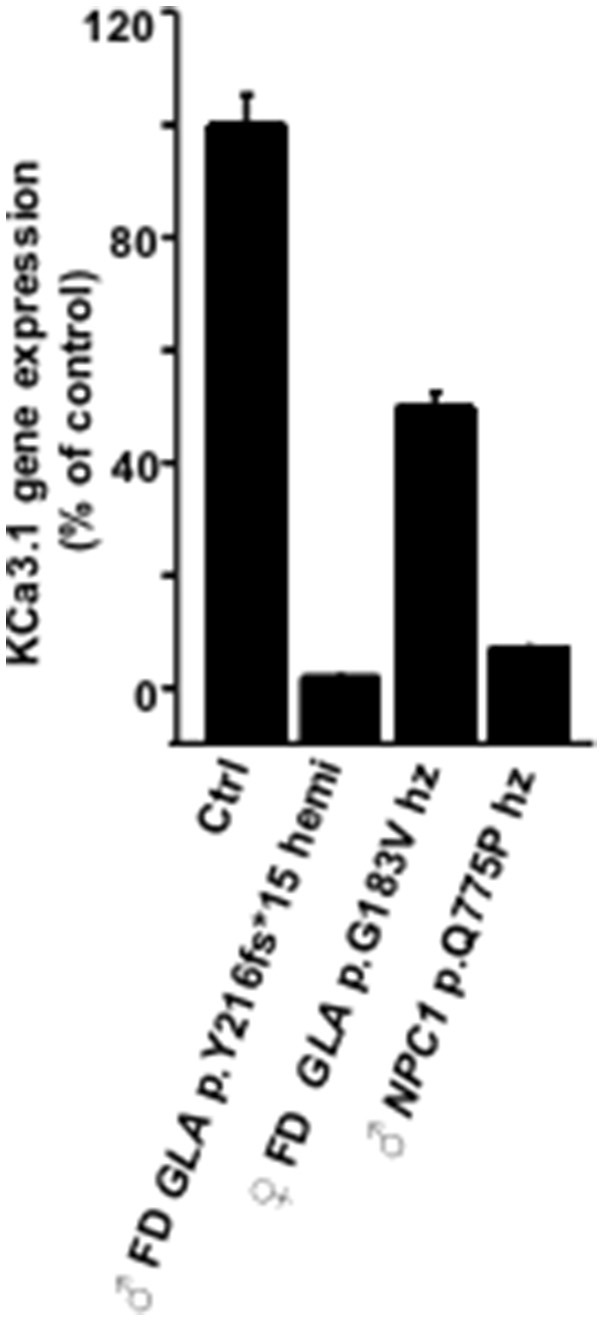
**Comparison of KCa3.1 gene expression and KCa3.1 current densities in fibroblasts from FD and NPC patients**. Results from qRT-PCR showing gene expression of KCa3.1 m in FD and NPC patients relative to control (Ctrl). Data are means of triplicates ± SEM.

Here, our mRNA-expression studies revealed substantial lower mRNA-KCa3.1 amounts in cell extracts from the male hemizygous FD-p.Y216fs^*^15 patient (Figure 4), the male NPC patient, but not from the female heterozygous FD-p.G183V patient.

**Figure 5:**
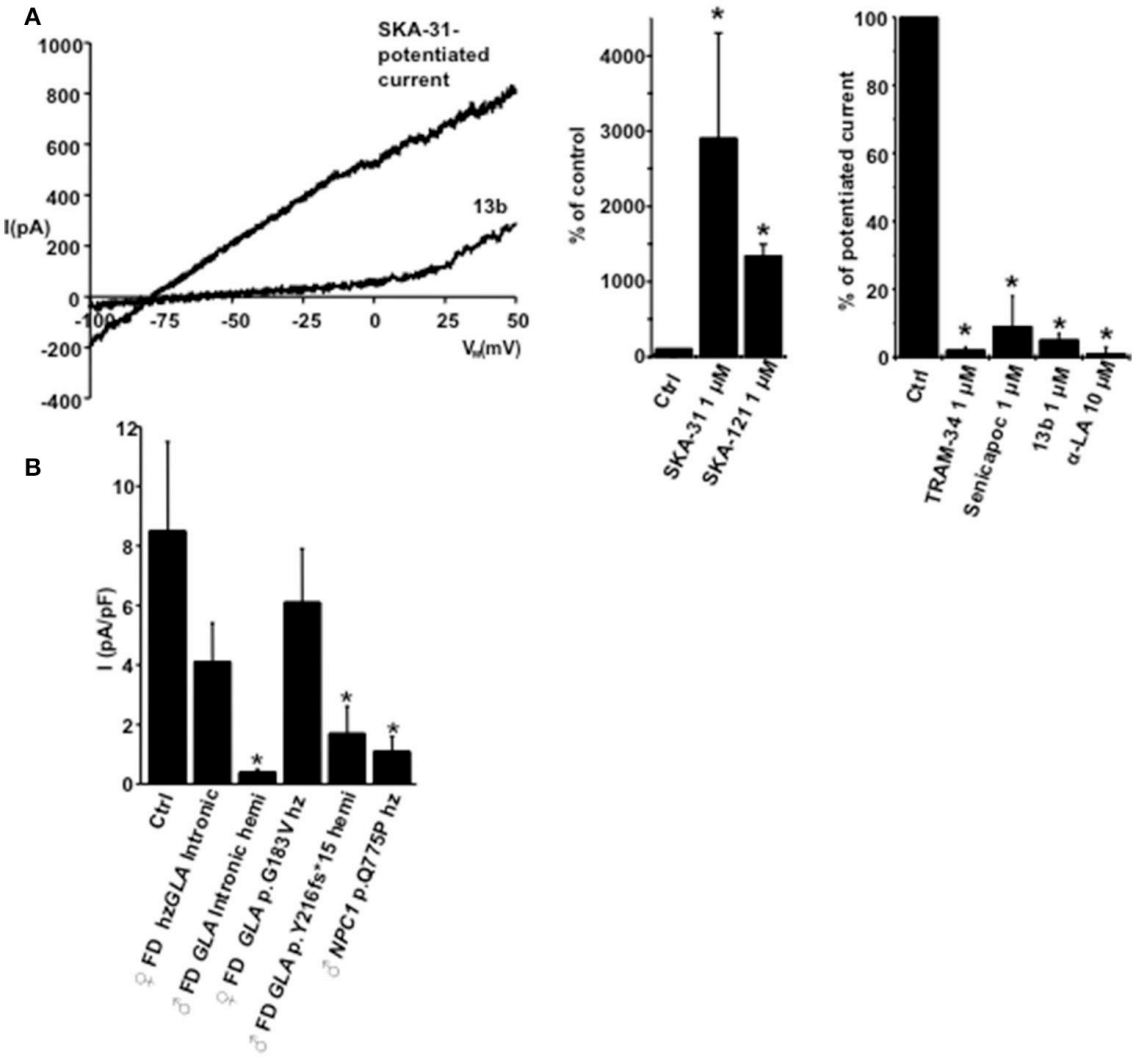
**Pharmacological characteristics of KCa3.1 in human fibroblasts. (A)** On left: Exemplary traces showing the SKA-31 (1 μM)-potentiated fibroblast KCa3.1 current and inhibition of the complete SKA-31-potentiated KCa3.1-current by 13b (1 μM). In middle: Potentiation of K currents (background K currents or calcium-pre-activated by calcium) by SKA-31 (*n* = 4) and SKA-121 (*n* = 5). On right: Inhibition of the SKA-potentiated current by TRAM-34 (*n* = 4), Senicapoc (*n* = 2), 13b (*n* = 10), and α-LA (*n* = 2). **(B)** Mean SKA-potentiated KCa3.1-current densities in Ctrl, FD, and NPC patients. Percentage of cells displaying functional KCa3.1: Ctrl, 4 out of 4 cells (100%); ♀ FD *GLA* Intronic heterozygous, 13 out of 20 (65%); ♂ FD *GLA* Intronic hemizygous 1 out of 15 (7%); ♀ FD *GLA* p.G183V heterozygous, 5 out of 6 (83%); ♂ FD *GLA* p.Y216fs^*^15 hemizygous, 4 out of 6 (67%); ♂ *NPC1* p.Q775P heterozygous, 3 out of 5 (60%). Data in bar chart are means ± SEM, ^*^P < 0.05, Student's *T*-test.

Our patch-clamp studies detected KCa3.1 currents in human control fibroblasts with the same biophysical and pharmacology profile as in 3T3-L1 fibroblasts and of cloned human KCa3.1, i.e., calcium-activation, potentiation by SKA-31 (29-fold) or by SKA-121(13-fold), a related, more selective KCa3.1 activator (Coleman et al., 2014; for traces and summary data see Figure 5A). This potentiated current was virtually abolished by TRAM-34, Senicapoc, the negative-gating modulator, 13b (Olivan-Viguera et al., 2013), and α-LA (Figure 5A, right panel).

The comparison of SKA-potentiated and maximal KCa3.1-currents densities in the LSD patients revealed a clear trend toward lower functional KCa3.1 expression in the two hemizygous male FD patients and the male NPC patient but not in heterozygous female FD patients (Figure 5B): Mean KCa3.1 current densities were reduced to 5 and 20% of control in the two male FD, to 48 and 72% of control in the two female heterozygous FD patients, and to 13% in the male NPC patient.

Together, our data suggests that KCa3.1-gene expression and membrane expression of the channel are impaired in fibroblasts of hemizygous male FD patients and in the male NPC patients, while KCa3.1-functions in fibroblasts from heterozygous female FD patients are largely conserved.

## Discussion

KCa3.1 channel functions have been linked to abnormal cell proliferation, pathological tissue remodeling and fibrosis of a variety of organs, chronic inflammation, and autoimmune diseases (Wulff and Zhorov, 2008; Roach et al., 2013; Wulff and Köhler, 2013; Feske et al., 2015; Huang et al., 2015; Köhler et al., 2016). Accordingly, pharmacological inhibition of KCa3.1 has been suggested to be a treatment strategy for such disease states. In the present study we investigated: (1) the modulation of fibroblast KCa3.1 channels and fibroblast proliferation by omega-3 fatty acids, α-LA and DHA, as well as α-LA/γ-LA-rich and -low plant oils. (2) KCa3.1 functions in FD and NPC fibroblasts.

Here, we demonstrated KCa3.1 functions with the typical pharmacological fingerprint in murine 3T3-L1 fibroblasts and primary human dermal fibroblasts and showed inhibition of the channel by α-LA and DHA as well as potent anti-proliferative effects of these omega-3 fatty acids *in vitro*. Moreover, we demonstrated inhibition of KCa3.1 functions by α-LA- and γ-LA-rich plant oils and strong inhibition of 3T3-L1 fibroblast by linseed oil and borage oil, suggesting them as non-synthetic channel inhibitors with potential utilities for alleviating diseases states characterized by excessive cell proliferation, fibrosis, and chronic inflammation.

Impaired KCa3.1 functions have also been suggested to be a result of lipid overload in FD (Choi et al., 2014). Here, we demonstrated conserved but significantly reduced functional KCa3.1 channel membrane expression and mRNA expression in classical and atypical male FD patients and a NPC patient, KCa3.1 functions, suggesting impaired expression of KCa3.1 as potential biomarker of these LSDs.

Lipid regulation of K channels is a rather new field in ion channel research. So far, activation as well as inhibition of K channels has been described: Omega-6 and -3 fatty acids have been reported to activate KCa1.1 channels (a.k.a. BKCa encoded by the KCNMA1 and KCNMB1-3 genes; Kirber et al., 1992; Wei et al., 2005; Moreno et al., 2012). Likewise, the K2P channels, TWIK-2/2, TREK1/2, and TRAAK, were activated by omega-3/6 acids (Maingret et al., 1999; Wei et al., 2005; Nielsen et al., 2013). Regarding the KCa2/3 gene family, cloned KCa3.1 was, however, inhibited by omega-3/6 fatty acids (Hamilton et al., 2003; Kacik et al., 2014) and this inhibition required the inner-cavity lining amino acid residues T250 and V275 that are not present in corresponding sites in the above mentioned K channels families and in KCa2 channels. In the present study we provided new evidence for KCa3.1-inhibition by α-LA and also showed for the first time that also α-LA/γ-LA-containing plant oils (at 0.5%) were capable of efficiently inhibiting KCa3.1. However, inhibitory activity clearly depended on high α-LA or γ-LA-content (>30%) since oils with low α-LA/γ-LA content failed to produce significant inhibition, which could be explained by the high contents of saturated or mono-saturated fatty acids that were not active on KCa3.1 but competed with α-LA as suggested previously for P450-derived metabolites of arachidonic acid such as 5,6-EET, 8,9-EET, 5,6-DiHETE, and saturated arachidic acid (Kacik et al., 2014).

The possible consequences of KCa3.1-inhibtion by α-LA for fibroblast proliferation were not established. Here, our study extended the current knowledge by showing that the α-LA- and γ-LA-rich plant oils, linseed oil and borage oil, respectively, exerted strong anti-proliferative activity. The γ-LA-low onagra oil was inefficient, a finding that further fostered the notion that for efficient targeting KCa3.1 and inhibition of cell proliferation a high-α-LA-/γ-LA was needed. Concerning argan oil, we cannot judge on possible anti-proliferative actions because the oil was found to be cytotoxic at the concentration used here.

Concerning the magnitude of inhibition of fibroblast proliferation the effect of α-LA and α-LA-rich oils were similar to reported effects of a synthetic negative-gating modulator of KCa3.1 and a classical pore blocker (Olivan-Viguera et al., 2013; Roach et al., 2015). However, here we do not wish to exclude other mechanisms by which α-LA and α-LA-/γ-LA-rich oils reduce fibroblast proliferation.

Dietary supplementation with α-LA and α-LA-containing oils has been suggested since long to have therapeutic utilities in several autoimmune diseases of skin and bowel, multiple sclerosis, and rheumatoid arthritis (for review see: Macfarlane et al., 2011; Wergeland et al., 2012). Some of these effects have been proposed to rely on interference with e.g., pro-inflammatory prostaglandin production/metabolism (for review see Yates et al., 2014).

Here, we suggest that inhibition of KCa3.1 could be an additional mechanism of action. KCa3.1 inhibitors have been shown to suppress pro-inflammatory cytokine production in immune cells and to reduce pathological organ remodeling and fibrosis in experimental models. Therefore, from the perspective of phytopharmacology, our data fostered the view that inhibition of KCa3.1 in psoriatic lesions of skin or the chronically inflamed bowel by topically or systemically applied α-LA/γ-LA-rich plant oils and/or dietary supplementation with α-LA/γ-LA-rich plant oils or by synthetic blockers (Wulff and Köhler, 2013; Christophersen and Wulff, 2015) could be an alternative and economic treatment for patients with mild forms of disease or an adjuvant treatment in more severely affected patients receiving immune suppressive therapy.

It should also be considered that the plant oils used in this study are of complex composition and the nature of the active compounds is still unclear. So, we do not wish to exclude that other compounds than α-LA caused some of the anti-proliferative effects on 3T3 fibroblasts. Yet, it was striking that the anti-proliferative effects correlated with the quantity of α-LA or γ-LA suggesting that α-LA/γ-LA played a major role here. In this regard, it is worth speculating that the cytotoxic effects caused by argan oil likewise rely on so far undefined compounds in this oil.

Dysfunction of lysosomal lipid metabolism/transport has recently been shown to impair KCa3.1-functions and mRNA-expression and reduced membrane surface expression by lowered phosphatidylinositol 3-phosphate as reason for these alterations (Choi et al., 2015; Choi and Park, 2016). In the present study, we provided additional evidence for such defective KCa3.1-regulation in FD fibroblasts and also in NPC fibroblasts from a male patient, which seemed predominantly to rely on impaired *de novo*-KCa3.1 gene expression, thus similar to that was found in earlier studies (Choi et al., 2015; Choi and Park, 2016). To the contrast, membrane expression—albeit at lower density—and activation was still intact in most cells, suggesting no major disturbances in membrane trafficking or function in the fibroblasts from FD patients investigated in our study.

Interestingly, the alterations were exclusively present in dermal fibroblasts of male FD patients with a classical missense mutation of this X-linked disease and an atypical complex intronic haplotype that have been reported to produce FD pathologies. In contrast, heterozygous female carriers with classical and atypical mutations, who may or may not develop clinically relevant symptoms depending on whether they have the mutated allele or the healthy allele as active copy, showed largely conserved functions of the channel in their dermal fibroblasts. Together, this suggested that low KCa3.1-mRNA expression in skin fibroblasts could be at least an additional biomarker of FD-disease or disease activity in male FD patients and in NPC patients.

Interestingly, in another study on KCa3.1 functions in type-1 and -3 Gaucher patients we found a defect in the physiologically occurring up-regulation of KCa3.1 during monocyte-to-macrophage differentiation (Oliván-Viguera et al., 2015a), further suggesting that KCa3.1 dysregulation is perhaps a more general feature of LSDs and cellular pathophysiology. But again, the pathophysiological mechanism and significance remain unclear.

At this point, we would like to remind the reader that KCa3.1 functions in disease states have been so far related to pro-proliferative and -inflammatory processes and pathological organ remodeling such as kidney and lung fibrosis (Wulff and Zhorov, 2008; Roach et al., 2013; Wulff and Köhler, 2013; Feske et al., 2015; Huang et al., 2015; Köhler et al., 2016). Accordingly, pharmacological inhibition of KCa3.1 has been proposed as pharmacological strategy to treat chronic inflammation and organ fibrosis. So, whether or not KCa3.1—albeit reduced—is drug target in FD and associated pathologies such progressive heart and kidney disease, aneurism, and angiokeratoma cannot be decided at present. Still, there is the possibility that inhibition of KCa3.1 by small molecules or α-LA-/γ-LA-rich oils as dietary supplement may delay disease progression in LSD patients who develop organ pathologies characterized by chronic inflammation and fibrosis.

There are some limitations concerning our patient study: First, the number of patients studied here was relatively small, which is a general limitation when studying these rare LSD with complex and varying clinical pathology. However, the observed differences in KCa3.1-expression and functions were still considerable (ca. 75% reduction) between controls and male FD patients. Here additional, mRNA expression analysis in a larger number and other cohorts of patients will be needed to further establish KCa3.1 as biomarker of FD and/or other LSD. Whether these alterations were unique for FD disease remains unclear since a male NPC patient showed similarly reduced KCa3.1 functions. Secondly, in the present study we did not intent to reveal the pathomechanism linking defects in lipid metabolism and lipid overload to reduced KCa3.1 membrane functions, although lipid overload affecting *de novo*-gene expression is likely to be a major reason here.

In conclusion, the present study showed inhibition of KCa3.1 functions and fibroblast mitogenesis by the α-LA-/γ-LA-rich plant oils, *in vitro*. This suggests the utility of both as dietary supplement or topical ointments to target these pro-inflammatory and pro-mitogenic channels in cutaneous, neuronal or intestinal autoimmune disease.

## Author contributions

AO and RK conceived the study, conducted research, analyzed data, and wrote the manuscript. JL, LL, and ÁLG conducted research and analyzed data. EA, YG, PG, AJG, JC, PI, conducted research on patients, analyzed data, provided materials, and compounds, and contributed to the writing of the manuscript.

## Funding

This work was supported by the European Community (FP7-PEOPLE MC CIG ·”BrainIK,” to RK); Department of Industry and Innovation, Government of Aragon (GIPASC-B105 to ÁLG); and the Fondo de Investigacion Sanitaria, Instituto de Salud Carlos III (CB06/07/1036, to PG and RK).

### Conflict of interest statement

The authors declare that the research was conducted in the absence of any commercial or financial relationships that could be construed as a potential conflict of interest.
